# Detection of Life Threatening Ventricular Arrhythmia Using Digital Taylor Fourier Transform

**DOI:** 10.3389/fphys.2018.00722

**Published:** 2018-06-13

**Authors:** Rajesh K. Tripathy, Alejandro Zamora-Mendez, José A. de la O Serna, Mario R. Arrieta Paternina, Juan G. Arrieta, Ganesh R. Naik

**Affiliations:** ^1^Faculty of Engineering and Technology (ITER), Siksha ‘O’ Anusandhan Deemed to be University, Bhubaneswar, India; ^2^Electrical Engineering Faculty, Universidad Michoacana de San Nicolas de Hidalgo, Morelia, Mexico; ^3^Department of Electrical Engineering, Autonomous University of Nuevo León, Monterrey, Mexico; ^4^Department of Electrical Engineering, National Autonomous University of Mexico, Mexico City, Mexico; ^5^Sanatorio Güemes, Buenos Aires, Argentina; ^6^Biomedical Engineering and Neuromorphic Systems (BENS) Research Group, MARCS Institute, Western Sydney University, Penrith, NSW, Australia

**Keywords:** life threatening arrhythmia, Taylor-Fourier transform, magnitude and phase features, LSSVM, radial basis function kernel, classifier performance

## Abstract

Accurate detection and classification of life-threatening ventricular arrhythmia episodes such as ventricular fibrillation (VF) and rapid ventricular tachycardia (VT) from electrocardiogram (ECG) is a challenging problem for patient monitoring and defibrillation therapy. This paper introduces a novel method for detection and classification of life-threatening ventricular arrhythmia episodes. The ECG signal is decomposed into various oscillatory modes using digital Taylor-Fourier transform (DTFT). The magnitude feature and a novel phase feature namely the phase difference (PD) are evaluated from the mode Taylor-Fourier coefficients of ECG signal. The least square support vector machine (LS-SVM) classifier with linear and radial basis function (RBF) kernels is employed for detection and classification of VT vs. VF, non-shock vs. shock and VF vs. non-VF arrhythmia episodes. The accuracy, sensitivity, and specificity values obtained using the proposed method are 89.81, 86.38, and 93.97%, respectively for the classification of Non-VF and VF episodes. Comparison with the performance of the state-of-the-art features demonstrate the advantages of the proposition.

## 1. Introduction

**Figure 1:**
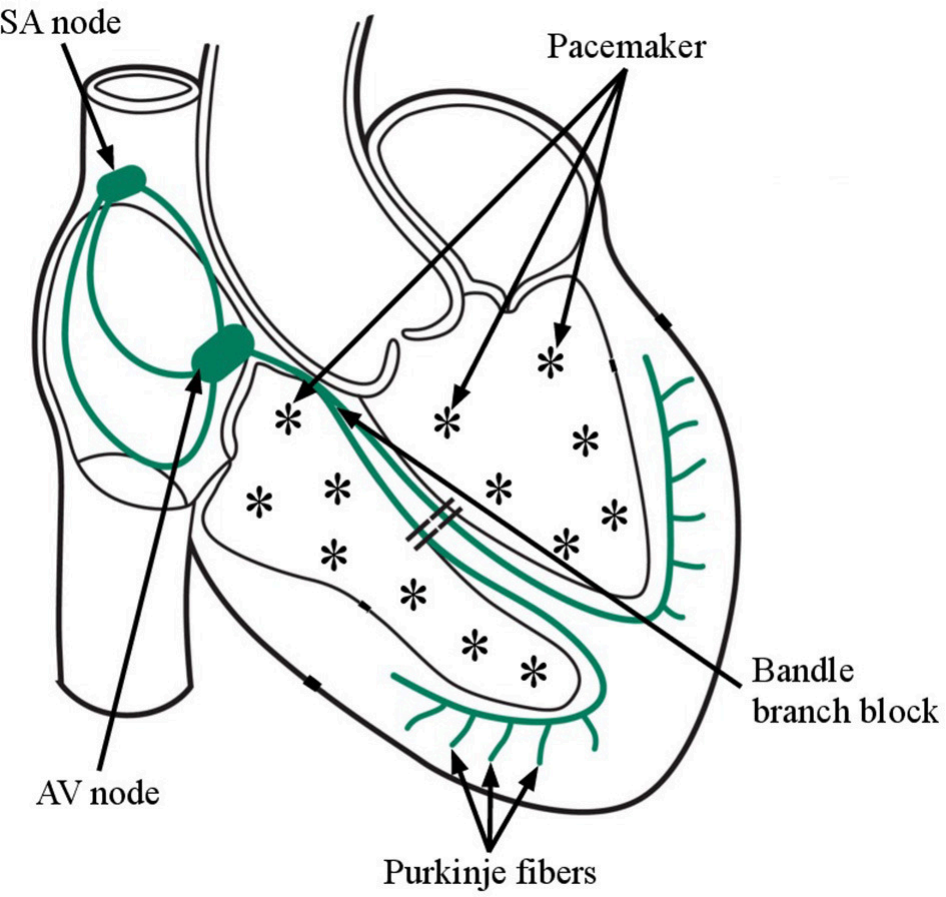
Firing of multiple pacemaker cells inside the lower chambers of the Heart during Ventricular Fibrillation (VF) as those from the normal pacemakers activity as (Sino atrial node- Atrio ventricular node-bundle of HIS- purkinje fibers.

The life threatening ventricular arrhythmias which require immediate defibrillation therapy are rapid ventricular tachycardia (VT) and ventricular fibrillation (VF) (Hunt et al., 2005; Acharya et al., 2018). The electrical activity of heart is no longer originated from sino-atrial node during these arrhythmias, rather it is started in the ventricular muscles which is shown in Figure 1 (Goldberger and Gold-berger, 1981). The pacemaker activity of heart is initiated from both left and right ventricles of the heart and due to this, the abnormal episodes other than the normal quasi-periodic PQRST components are observed in ECG signal (Goldberger and Gold-berger, 1981). The lower chambers of the heart such as the left and right ventricles are also ineffective to pump the blood to lungs and arteries. The defibrillation shock therapy is given to the patient affected with life threatening ventricular arrhythmia for recovering the normal heart rhythm (Tripathy et al., 2016). The detection and classification of shockable ventricular arrhythmia (VA) and non-shockbale episodes are the important and challenging problems in defibrillation therapy.

In literature, various methods have been reported for the detection and classification of life-threatening VA episodes using ECG (Jekova, 2000; Li et al., 2012; Acharya et al., 2018). One of the important step in the state of the art methods is the extraction of features from ECG signal for detection of VT/VF episodes. The most common features used for detection of VT and VF detection are based on complexity measure (CPLX) (Zhang et al., 1999), threshold crossing intervals (TCI) (Thakor et al., 1990), VF filter leakage measure (VFF) (), spectral algorithm (SPEC) (Barro et al., 1989), phase space representation (PSR) (Amann et al., 2007), autocorrelation function (ACF) (Chen et al., 1987), band-pass filter and count (Jekova and Krasteva, 2004), covariance, area and frequency bins of binary signal (Jekova, 2007), time-frequency analysis (Millet-Roig et al., 1999), and wavelet transforms (Addison et al., 2000; Balasundaram et al., 2013). The performance of five different VF detection algorithm has been compared in (Jekova, 2000). From their work, it has been found that the VF filter leakage measure has higher performance than other features. The combinations of aforementioned features of ECG have been used by Li et al. (2014) and Atienza et al. (Alonso-Atienza et al., 2014) for the detection of life-threatening VA episodes. In recent years, variational mode decomposition (VMD) and empirical mode decomposition (EMD) based analysis and extraction of features from ECG have been reported for detection of life threatening VA (Abdullah Arafat et al., 2009; Tripathy et al., 2016; Nguyen et al., 2017). Though, the VMD and EMD based techniques have better performance for detection, the real-time implementation of these algorithms require very higher computations. Therefore, a method which is computationally feasible and gives better performance for detection of life threatening VA is required.

The DTFT is one of the effective signal processing technique to decompose the non-stationary signal into oscillatory modes and it has been used for analysis of blood pressure and power signals (de la O Serna, 2007, 2013). Since, the characteristics of ECG signal varies during VF case as compare to normal heart rhythm, therefore, the DTFT can be used to capture these pathological changes in different oscillatory modes. The features extracted from the oscillatory modes of DTFT will be helpful for detection and classification of life-threatening episodes. The major contributions of this paper are highlighted as (i) Decomposition of ECG signal into oscillatory modes using DTFT. (ii) Evaluation of magnitude and phase features from the DTFT coefficients of each mode. (iii) The use of LSSVM classifier for detection and classification of shock vs. non-shock, VT vs. VF, and VF vs. non-VF episodes. The remainder of this paper is arranged as follows. The proposed method for detection of life threatening arrhythmia episodes is presented in section 2. The results and the discussion of the results are written in the sections 3, 4, respectively. The conclusion of this paper is drawn in section 5.

## 2. Method

**Figure 2:**
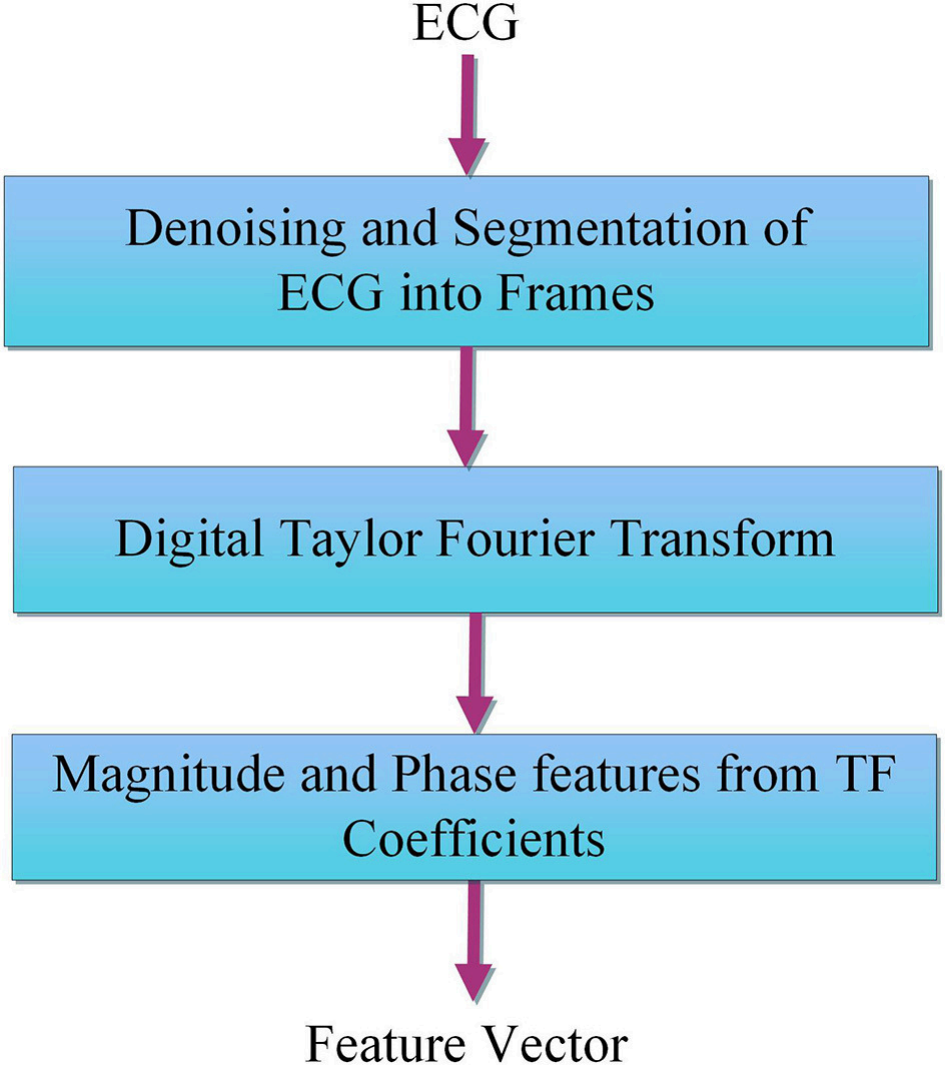
Block diagram of the proposed method for evaluation of Taylor-Fourier magnitude and phase features from ECG.

The proposed method consists of four major steps such as (i) ECG data collection, (ii) Preprocessing of ECG data, (iii) DTFT based feature extraction, and (iv) LSSVM based classification of life threatening arrhythmia episodes. The preprocessing and feature extraction step of the proposed method is shown in Figure 2.

### 2.1. ECG data collection from database

The ECG data are collected from two public databases namely the creighton university ventricular tachy-arrhythmia database (CUDB)^1^ and MIT-BIH malignant ventricular arrhythmia database (VFDB)^2^ (Goldberger et al., 2000). The CUDB database has 35 number of 8 min duration ECG signals with annotations as VT, VF, and other rhythms. Similarly, the VFDB database has 22 number of 35 min duration two lead ECG signals with annotations as NSR, VF, VT, ventricular flutter, and other rhythm. The sampling frequency of each ECG signal for both the databases is 250 Hz. In this study, three classification methodologies are considered namely shock vs. non-shock, VF vs. VT and VF vs. Non-VF. Under shock class, the ventricular flutter, VT and VF episodes are considered. Similarly, rhythms other than VF, VT, and ventricular flutter are taken under non-shock class. For non-VF class, the rhythms other than VF are considered. The purpose of selecting VF, VT, and ventricular flutter under shockable catagory is given as follows. The defibrillation has demonstrated to improve the outcome of patients suffering from cardiorespiratory arrest (CRA) due to VF, VT, or ventricular flutter (Wathen et al., 2004; Epstein et al., 2008). There are different rhythms of arrest, classified as defibrillable rhythms (such as VF) and no defibrillable rhythms (such as pulseless ventricular tachycardia). The main objective of the defibrillation therapy is to restore the spontaneous circulation, since the ventricular emptying is very compromised, enabling generate a hemodynamic collapse (Epstein et al., 2008). A common initial rhythm in the sudden cardiac arrest is VF, whose treatment consists of defibrillation. In this way, VT, VF, and ventricular flutter are defibrillable rhythms because they degenerate or lead to circulatory collapse. Thus they must be reversed promptly through defibrillation. In turn, this hemodynamic collapse produces a reduction in the transport of oxygen to the cell. Consequently an anaerobic metabolism (without oxygen) occurs (Wathen et al., 2004). In literature, for classification of shock vs. non-shock, authors have used VT, VF, and ventricular flutter as shock category and other ECG episodes as non-shockable rhythm (Alonso-Atienza et al., 2014; Acharya et al., 2018).

### 2.2. Preprocessing

This step involves the filtering of various noises from the ECG signals from CUDB and VFDB databases. A Zero-phase Butter-worth band-pass filter with cutoff frequencies as 0.5 and 45 Hz is used (Tripathy et al., 2016). The filtered ECG data is then divided into frames using three different rectangular windows (Li et al., 2014). The window sizes are 4, 5, and 8 s, respectively. As the sampling frequency of each ECG signal is 250 Hz, so the number of samples contained in the 4, 5, and 8 s windows are 1,000, 1,250, and 2,000, respectively. In this work, 5, 186, 4, 144, and 2, 582 number of 4, 5, and 8 s ECG frames are evaluated for the classification shockable VA and non-shockable episodes. Similarly, for the classification of VF and non-VF episodes, a total of 3432, 2744 and 1712 number of 4, 5, and 8 s ECG frames are computed. Likewise, 2, 593, 2, 072, and 1, 291 number of ECG frames are extracted for the classification of VT and VF episodes. This means that each ECG signal *x*(*n*) is partitioned into different frames of signals like **s**_1_, **s**_2_, ⋯ , **s**_*m*_, which will be processed by DTFT in the following.

### 2.3. DTFT based feature extraction

**Figure 3:**
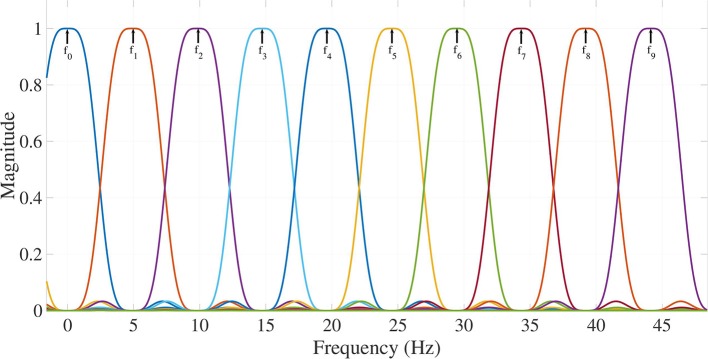
Frequency response of Taylor-Fourier filters exhibiting maximum flat differentiators and flat bands in harmonic frequencies for extracting 10 frequency components (Modes 0–9) from *f*_0_ (0.5 Hz) up to *f*_9_ (45 Hz).

From the phasor statement for the Taylor-Fourier Transform made in (de la O Serna, 2007) and the multivariate approach for low-frequency oscillations introduced in (Zamora et al., 2017), the DTFT technique has exhibited its reliability for extracting dynamic features in power systems. Here, we propose a Taylor-Fourier approach for capturing frequency information from biomedical signals like ECG signals. Our proposal also conceives the capability of the Taylor-Fourier filters for processing multiple frames, that is, a multiframes approach, allowing it to simultaneously deal with multiple frames stemming from first ECG lead, and render the estimated coefficients ($\hat{\xi }$) at the same time. To analyze the ECG signals using the Taylor-Fourier transform's multiframes approach, the synthesis and analysis equations, (1) and (2), respectively, are like in Zamora et al. (2017) for a set of *M* frames as follows:

(1) $$\begin{array}{l} \left[{\hat{\text{s}}}_{1}\;{\hat{\text{s}}}_{2}\;\cdots \;{\hat{\text{s}}}_{m}\right]=\text{B}\left[{\hat{\xi }}_{1}\;{\hat{\xi }}_{2}\;\cdots \;{\hat{\xi }}_{m}\right], \end{array}$$

(2) $$\begin{array}{l} \left[{\hat{\xi }}_{1}\;{\hat{\xi }}_{2}\;\cdots \;{\hat{\xi }}_{m}\right]={\text{B}}^{†}\left[{\text{s}}_{1}\;{\text{s}}_{2}\;\cdots \;{\text{s}}_{m}\right], \end{array}$$

where it is assumed that the ECG frames *s*_*m*_(*n*), *m* = 1, …, *M* contain 10 frequency components, as depicted in Figure 3. Each filter has a central frequency spaced 5 Hz, from 0 to 45 Hz. **B** stands for the Taylor-Fourier matrix in (de la O Serna, 2013) and **B**^†^ its pseudoinverse, i.e., the filter bank is assumed equal for all signals, covering all the spectral range for VF. These filters are employed for decomposing ECG frames signals into modes. **B** is shaped using Taylor and Fourier contributions as,

(3) $$\begin{array}{l} \text{B}=\left[\begin{array}{cccc} {\text{t}}_{n1}^{0} & {\text{t}}_{n1}^{1} & \cdots & {\text{t}}_{n1}^{K}\\ {\text{t}}_{n2}^{0} & {\text{t}}_{n2}^{1} & \cdots & {\text{t}}_{n2}^{K}\\ \vdots & \vdots & \ddots & \vdots \\ {\text{t}}_{nC}^{0} & {\text{t}}_{nC}^{1} & \cdots & {\text{t}}_{nC}^{K} \end{array}\right]\left[\begin{array}{cccc} {\text{W}}_{N} & 0 & \cdots & 0\\ 0 & {\text{W}}_{N} & \cdots & 0\\ \vdots & \vdots & \ddots & \vdots \\ 0 & 0 & \cdots & {\text{W}}_{N} \end{array}\right] \end{array}$$

where *C* = *K*+1 is the number of cycles and **W**_*N*_ corresponds to the Fourier matrix as $\omega_{N}=\left[e^{j2\pi nk/N}\right]$ with *n, k* = 0, …, *N*−1. **t_n_** = −(*K*+1)*T*_*s*_(*n*_*s*_/2) to (*K* + 1)*T*_*s*_(*n*_*s*_/2), *n*_*s*_ corresponding to each sample of the Taylor's interpolating polynomial at each sampling time (*T*_*s*_). Thus, the dimension for ${\text{t}}_{nC}^{K}$ in (3) is *N*x*N*, so that the dimension for the Taylor contribution is *CN*x*CN*; likewise the dimension for the Fourier contribution is *CN*x*CN*. That is, the vectors of the Fourier matrix are harmonic modulators of the Taylor terms included in a *K* − *th* Taylor polynomial, *K* > 0.

The parameters such as the magnitude and phase for *j*-th frequency component or mode are evaluated using Equation (2) and these are given by

(4) $$\begin{array}{l} {\hat{\text{a}}}_{j}=|{\hat{\xi }}_{j}| \end{array}$$

(5) $$\begin{array}{l} {\hat{\phi }}_{j}=∠{\hat{\xi }}_{j}. \end{array}$$

**Figure 4:**
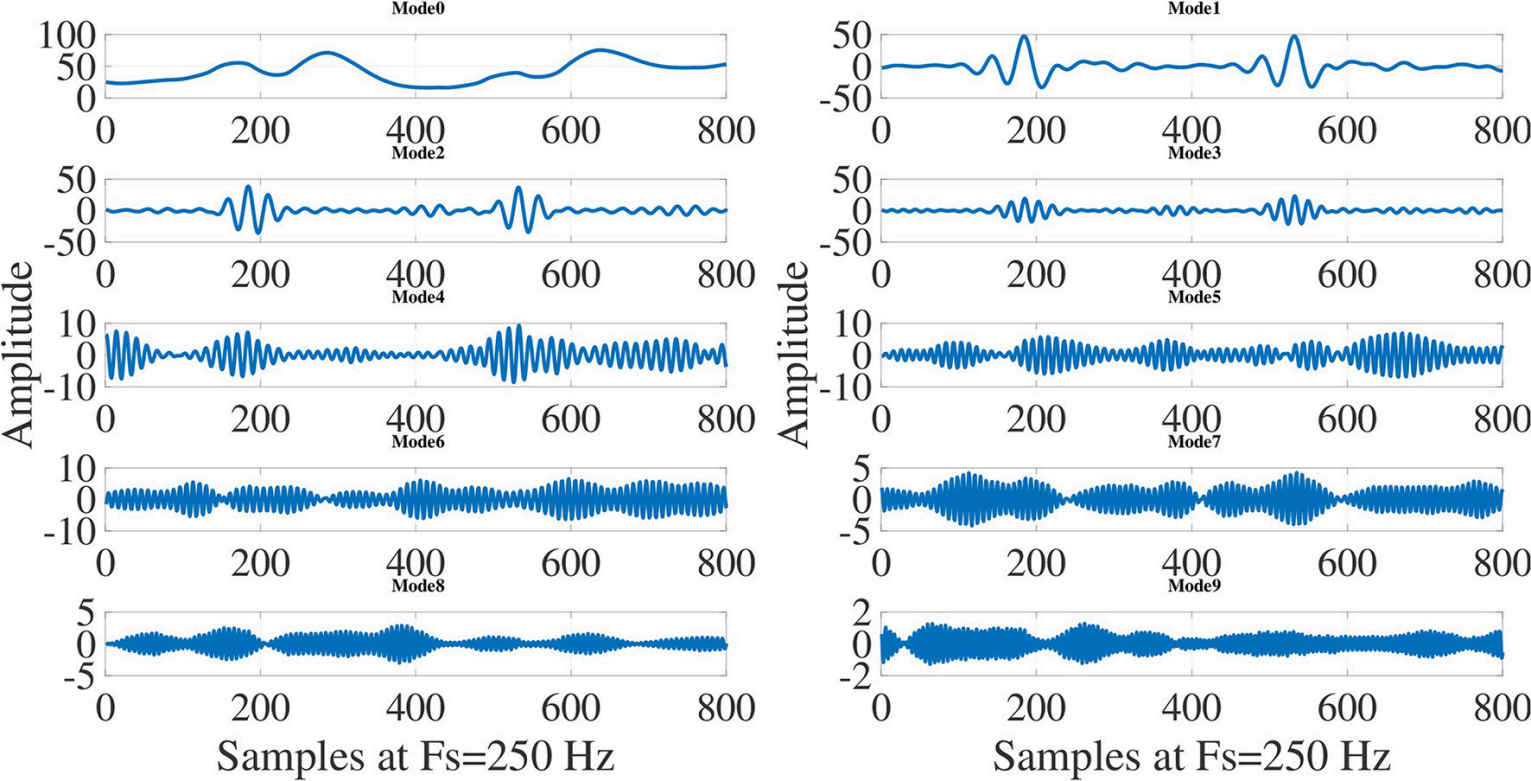
Decomposition of normal sinus rhytm (NSR) ECG signal into modes using Taylor-Fourier filter bank and first 10 modes (Mode 0 to Mode 9) of NSR ECG signal.

**Figure 5:**
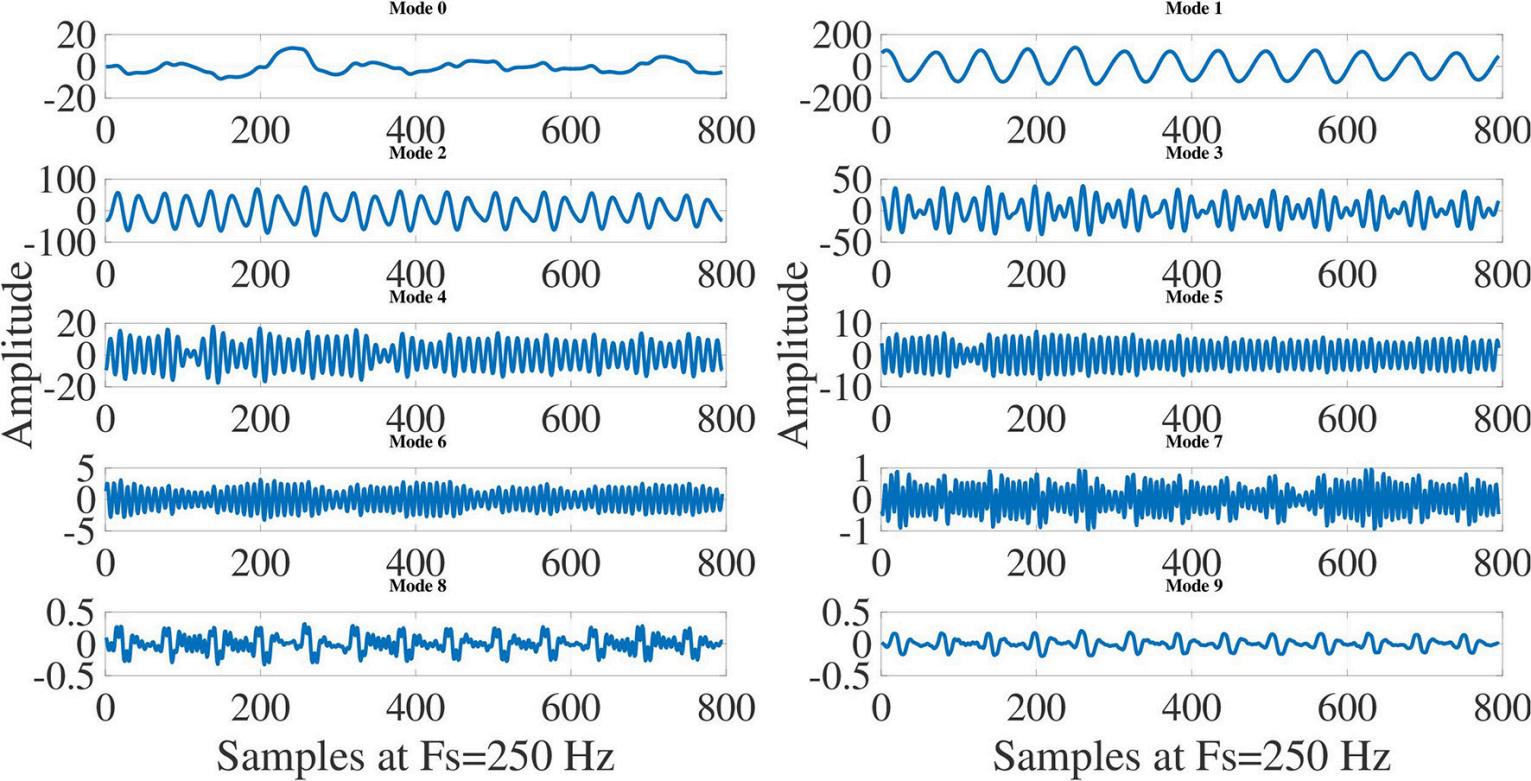
Decomposition of ventricular tachycardia (VT) ECG signal into modes using Taylor-Fourier filter bank and first 10 modes (Mode 0 to Mode 9) of VT ECG signal.

**Figure 6:**
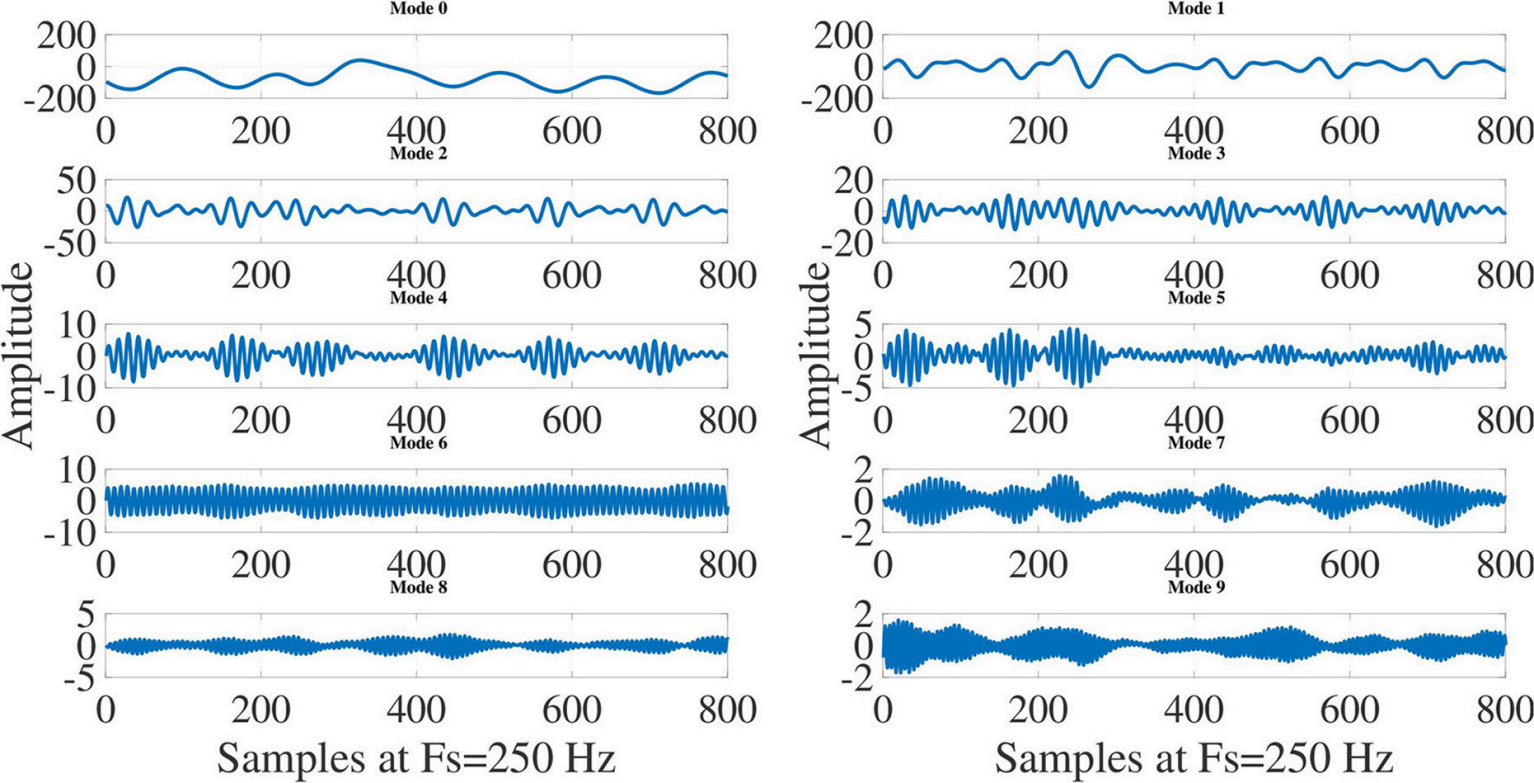
Decomposition of ventricular fibrillation (VF) ECG signal into modes using Taylor-Fourier filter bank and first 10 modes (Mode 0 to Mode 9) of VF ECG signal.

The 10-mode decompositions (Mode 0 to Mode 9) of ECG signals for normal sinus rhythm (NSR), VT and VF cases are depicted in Figures 4, 5, 6, respectively. These modes are reconstructed from the Taylor-Fourier coefficients using Equation (1). As it is evident from these three figures that the characteristics of each mode is different for NSR, VT, and VF cases. From Figure 4, it has been observed that, the information of QRS-complex can be grossly captured using Mode 1 to Mode 6; moreover, it can be distinguished a sinus rhythm and regular with PR impresses normal, a narrow QRS, and an iso-level ST. Whereas the abnormal patterns other than the grossly segregated QRS-patterns are observed in each mode of VT and VF ECG signal (Figures 5, 6), among them, it is observable that the electrocardiographic tracing presents a wide QRS tachycardia, with atrio-ventricular dissociation, compatible with sustained polymorphous VT of approximately 105 beats per minute (BPM), which implies that in 8 s there are 14 beats (Goldberger and Gold-berger, 1981). Thus, the features extracted from these modes can be helpful for detection of life threatening ventricular arrhythmia. In this work, the magnitude feature such as the *L*^2^-Norm of the Taylor-Fourier coefficients and a novel phase feature such as phase difference (PD) are extracted from each mode of ECG signal. The magnitude feature of the *j*-th mode (*j* = 1, 2, …, *M*) is given by

(6) $$\begin{array}{l} MF_{j}=||{\hat{\text{a}}}_{j}|{|}_{2} \end{array}$$

where ${\hat{\text{a}}}_{j}=\left[{â}_{j}\left(1\right),{â}_{j}\left(2\right),\ldots ,{â}_{j}\left(N\right)\right]$. The PD feature for of the *j*-th frequency component or mode is evaluated in two steps as (i) the evaluation of phase delay vectors as $d1_{j}=\left[{\hat{\phi }}_{j}\left(1\right),{\hat{\phi }}_{j}\left(2\right),\ldots ,{\hat{\phi }}_{j}\left(K-1\right)\right]$ and $d2_{j}=\left[{\hat{\phi }}_{j}\left(2\right),{\hat{\phi }}_{j}\left(3\right),\ldots ,{\hat{\phi }}_{j}\left(K\right)\right]$, respectively. The PD is defined by

(7) $$\begin{array}{l} PD_{j}=\frac{1}{K-1}\overset{K-1}{\underset{l=1}{\sum }}|d1_{j}\left(l\right)-d2_{j}\left(l\right)| \end{array}$$

For all the 10 modes of ECG, the magnitude and the PD features are evaluated. Thus, a 20 dimensional Taylor-Fourier feature vector is constructed by appending the magnitude and PD features.

**Figure 7:**
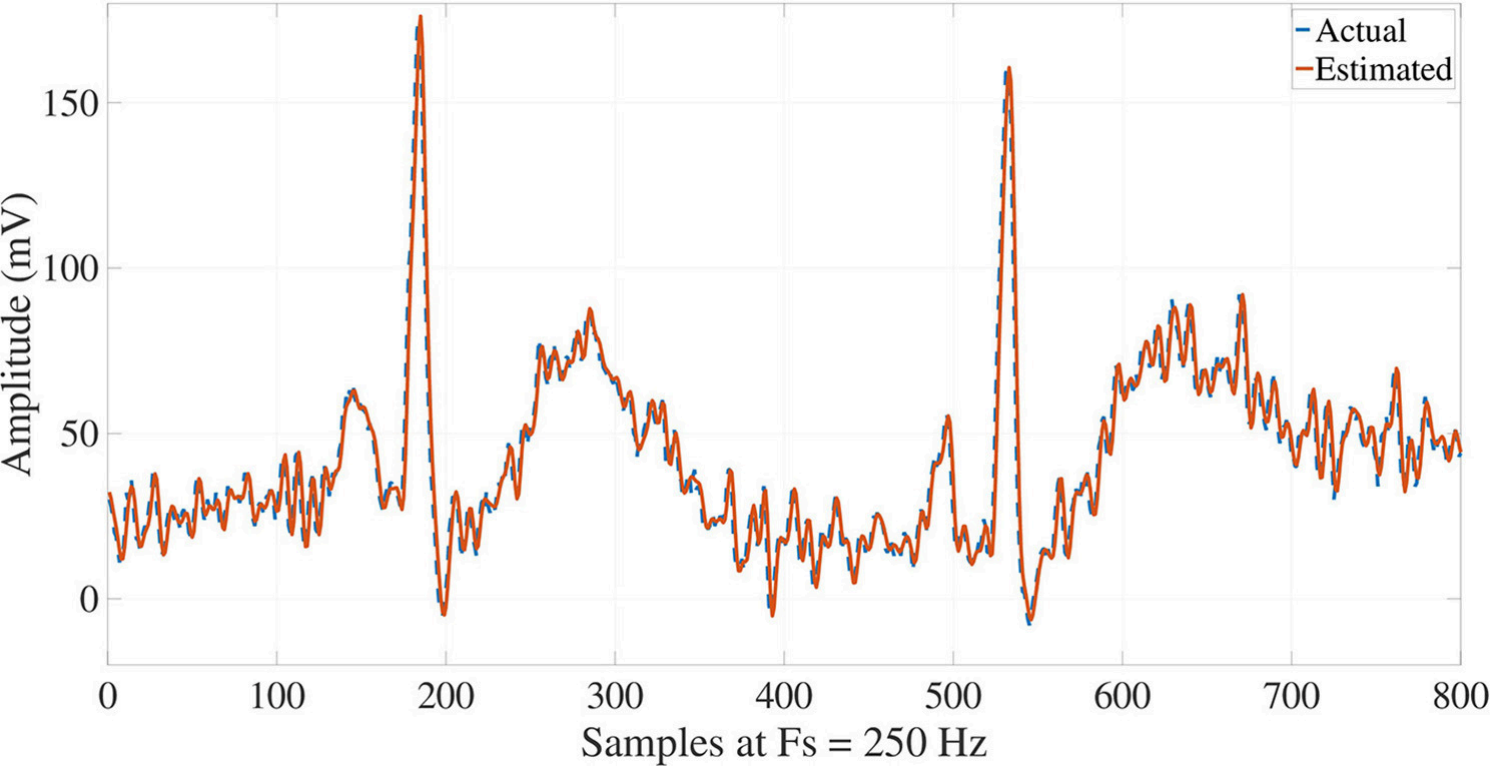
Reconstruction of NSR ECG signal from the Taylor-Fourier coefficients.

**Figure 8:**
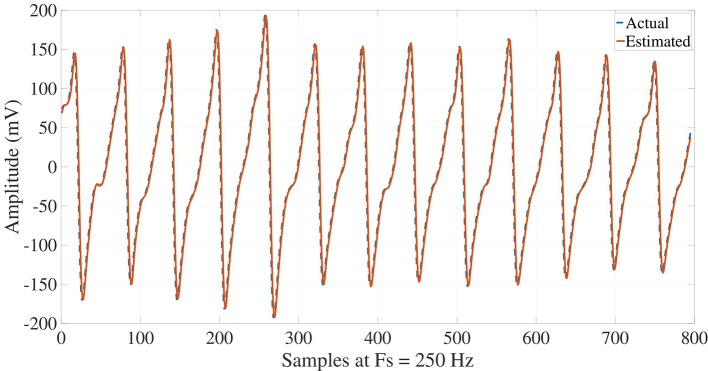
Reconstruction of VT ECG signal from the Taylor-Fourier coefficients.

**Figure 9:**
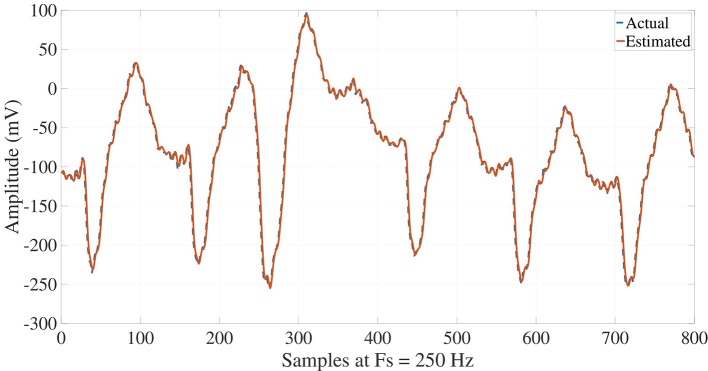
Reconstruction of VF ECG signal from the Taylor-Fourier coefficients.

Once the ECG signals are processed, their reconstructions may carry out synthesizing all the modes by Equation (1), such that a suitable performance and matching are attained with respect to the actual signals for NSR, VT and VF signals, as illustrated in Figures 7, 8, 9. NSR case in Figure 7 presents a sinus rhythm, regular, normal PR, narrow QRS, and iso-level ST. In the VT case in Figure 8, a sustained monomorphic ventricular tachycardia with a wide QRS, and more than 100 BPM is illustrated. Whereas VF case exhibits a shockable behavior (see Figure 9), in which an electrocardiographic tracing is observed with a wide-QRS tachycardia with sustained polymorphic VT with a very high rate. This result demonstrates the ability of the Taylor-Fourier filters for extracting frequency components and reconstructing the oscillatory signals still under hemodynamic unstability.

### 2.4. LSSVM classifier

In this work, the effectiveness of the magnitude and phase features from the mode Taylor-Fourier coefficients of ECG is assessed using LSSVM classifier for detection and classification of life threatening arrhythmia episodes. The objective of LSSVM is to evaluate the optimal weights and the bias value by formulating a least square problem (Suykens and Vandewalle, 1999). It has been used for various bio-medical applications such as detection of epileptic seizure, detection of breast cancer and detection of various cardiac arrhythmia episodes (Polat et al., 2008; Bajaj and Pachori, 2012; Tripathy et al., 2014). The classification of life threatening VA is performed using the 20 dimensional Taylor-Fourier feature vector. The feature matrix and the respective class labels are denoted as $\text{Z}={\left[{\text{z}}_{i}\right]}_{i=1}^{m}$ with each ${\text{z}}_{i}\in R^{p}$ and $\text{y}={\left[y_{i}\right]}_{i=1}^{m}$ with each *y*_*i*_ = (0, 1). Here, *m* is the total number of instances and 0 and 1 are the notations for Non-VF and VF ECG feature instances in the classification of VF vs. Non-VF. Similarly, for the classification of VT vs. VF, 0 and 1 are the class labels for VT and VF classes. Likewise, the 0 and 1 are also termed as the class labels for non-shock and shock classes for the classification of shock vs. non-shock. The optimization problem in LSSVM is given by (Suykens and Vandewalle, 1999)

(8) $$\begin{array}{l} Minimize\;J\left(\text{w},b,\epsilon \right)=\frac{1}{2}{\text{w}}^{T}\text{w}+\frac{\gamma }{2}\overset{p}{\underset{i=1}{\sum }}\epsilon_{i}^{2} \end{array}$$

subjected to the equality constraint as $y_{i}\left({\text{w}}^{T}f\left({\text{z}}_{i}\right)+b\right)=1-\epsilon_{i}$. where **w** and *b* are the q-dimensional weight vector and bias value, respectively. The function *f*(**z**_*i*_) maps the input p-dimensional feature vector into a q-dimensional space. The above equation can be solved using Lagrangian as

(9) $$\begin{array}{l} L\left(\text{w},b,\epsilon ;\beta \right)=J\left(\text{w},b,\epsilon \right)-\overset{m}{\underset{i=1}{\sum }}\beta_{i}\left[y_{i}\left({\text{w}}^{T}f\left({\text{z}}_{i}\right)+b\right)-1+\epsilon_{i}\right] \end{array}$$

The solution of Equation (9) will give rise to the corresponding Lagrange multipliers as $\beta ={\left(\beta_{1},\beta_{2},\ldots ..,\beta_{m}\right)}^{T}$. Thus, the output of LSSVM classifier for a given test ECG Taylor-Fourier feature vector **z**_*t*_ can be written as

(10) $$\begin{array}{l} f\left({\text{z}}_{t}\right)=sign\left[\overset{m}{\underset{i=1}{\sum }}\beta_{i}y_{i}K\left(\text{z},{\text{z}}_{i}\right)+b\right] \end{array}$$

where the term *K*(**z**, **z**_*i*_) is denoted as the kernel function. Here, the linear and RBF kernel functions are used and the classification performance of LSSVM with these two kernel functions are compared. The training and testing Taylor-Fourier feature vectors of ECG frames are chosen using both hold-out and 5-fold cross-validation approaches (Martis et al., 2013). In hold-out approach, 65% of Taylor-Fourier feature vectors from the feature matrix **Z** is used for training and the rest of the Taylor-Fourier feature vectors are considered for testing of LSSVM. The performance of LSSVM is evaluated using the measures such as the accuracy, sensitivity and specificity (Tripathy et al., 2016).

## 3. Results

**Figure 10:**
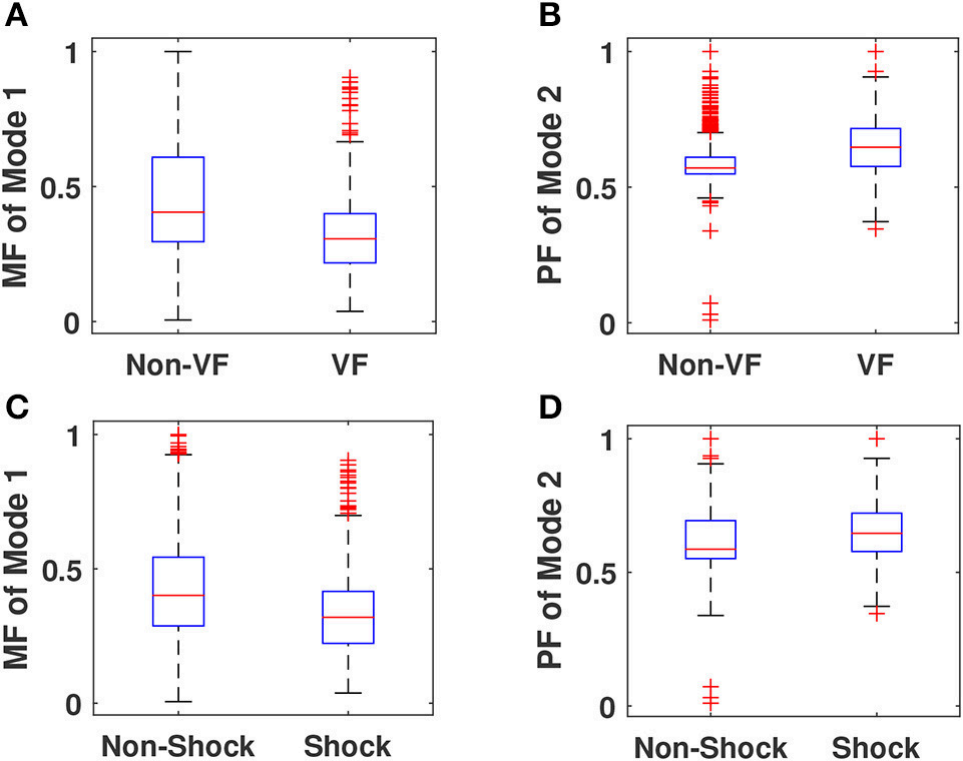
**(A)** Within-class variations of magnitude feature (MF) of mode1 for VF and non-VF classes. **(B)** Within-class variations of phase feature (PF) of mode 2 for VF and non-VF classes. **(C)** Within-class variations of magnitude feature (MF) of mode 1 for shockable VA and non-shockable classes. **(D)** Within-class variations of phase feature (PF) of mode 2 for shockable VA and non-shockable classes.

This section presents the statistical analysis results of Taylor-Fourier magnitude and phase features of ECG signal and the performance of LSSVM classifier. The within-class variations of selected Taylor-Fourier magnitude features are shown in boxplots as in Figures 10A,C, respectively. From Figure 10A, it is evident that the mean value of magnitude feature of mode1 is higher for non-VF class as compared to VF class. From the modal decomposition of Non-VF and VF ECG signals in Figures 4, 6, it has been observed that there are the significant variations in the characteristics of mode1. Due to this reason, the mean values are different for both Non-VF and VF classes. It is also seen that, for shockable class the magnitude feature of mode1 has higher mean value than that of non-shockable class. Similar variations in the mean values has been observed for magnitude features of selected modes. The ECG signal for non-shockable class (NSR) contains the normal clinical patterns such as the P-wave, QRS-complex, and T-wave for each beat (Goldberger and Gold-berger, 1981). The pathological patterns with a very high rate are observed in ECG during VF and rapid VT cases. Also, the amplitudes and shapes of these patterns are different as those of the normal clinical patterns in non-shockable cases (Clifford et al., 2006). From Figures 4, 6, it can also be observed that the information about the pathological patterns due to VF is captured in the modes which are captured using different resolutions for the Taylor-Fourier coefficients. As the Taylor-Fourier magnitude features are evaluated from these coefficients, these features have different mean values for both shockable and non-shockable classes. The variations of phase features of mode2 for non-shock vs. shock and non-VF vs. VF classes are depicted in Figures 10B,D, respectively. For shockable class, the phase feature has higher mean value as compared to non-shock class. Similar variation in the mean values is also seen for non-VF and VF classes. The sub-band phase features evaluated using the complex wavelet transform of ECG have been used for quantifying physiological changes into ECG signal during myocardial infarction and other pathologies (Tripathy and Dandapat, 2017). In this work, the temporal information of ECG is captured using the Taylor-Fourier phase features of different modes. In VF and rapid VT pathologies, the temporal variations in ECG signal are higher as compared to the normal heart rhythm (Goldberger and Gold-berger, 1981). Since this reason, the phase features have higher mean value for VF class. The statistical significance of all Taylor-Fourier magnitude and phase features are evaluated using *t*-test approach. It is observed that for the classification of VF and non-VF, only 17, 17, and 14 features have *p*-values less than 0.001 out of 20 dimensional feature vectors of 4, 5, and 8 s ECG frames. Similarly, 16, 12, and 15 features out of the 20 dimensional feature vectors of each 4, 5, and 8 s ECG frames are statistically significant for the classification of shock and non-shock episodes. From the statistical analysis, it can be inferred that the Taylor-Fourier magnitude and phase features of ECG correctly capture the diagnostic information of ECG signal for detection of life threatening VA.

The performance of LSSVM for the classification of non-shock vs. shock, Non-VF vs. VF and VF vs. VT using the Taylor-Fourier magnitude and phase features of all databases, only VFDB database and only CUDB database are shown in Tables 1, 2, 3, respectively. It is evident that the accuracy, sensitivity, and specificity values of LSSVM for the classification of non-VF and VF episodes are 89.91, 86.38, and 93.97% using Taylor-Fourier magnitude and phase features of 8 s frames of ECG signals from VFDB database. The accuracy value of LSSVM classifier is higher using 8 s frame based ECG features as compared to 5 and 4 s frame based magnitude and phase features. The Taylor-Fourier magnitude and phase features of 8 s ECG frames in VFDB database effectively captures the pathological changes of ECG during VF. For CUDB database, the accuracy of LSSVM with Taylor-Fourier magnitude and phase features of 4 s frames of ECG signals is higher than those of the 5 and 8 s ECG frames. The performance of RBF kernel based LSSVM is higher than linear kernel by using the features of 4, 5, and 8 s frames of ECG signals of the combination of VFDB and CUDB databases. For the classification of VF vs. VT, the performance of proposed method using the ECG signals of CUDB database is higher than the VFDB database. The proposed method has the advantage that it can detect and classify life threatening VA episodes. The sensitivity and specificity values for the classification of VT and VF episodes using LSSVM classifier and the features of 4 s ECG frames are 82.41 and 95.44%, respectively. The ECG signal is widely used to quantify the pathological similarities and differences between ventricular tachycardia and ventricular fibrillation (Goldberger and Gold-berger, 1981). The electrocardiographic diagnosis of VT is carried out in the presence of three or more complex QRS, presenting an aberrant configuration and a ventricular origin (3 or more EV), whose cardiac frequency is 120 beats per minute or greater. Tachycardia may become regular or irregular, whereas the atrial activity can be independent of the ventricular-atrial dissociation, or it can be linked to VT by a reverse conduction 1:1 or a variable-degree heart block (Goldberger and Gold-berger, 1981). According to their electrocardiographic configuration, VTs are divided into uniform, biform or multiform. With respect to the ventricular fibrillation, this is characterized by a chaotic activation of myocardium. According to the mechanism for VF there exist different clinical conditions with the potential to yield it, such as: ischemia, acute myocardial infarction, drugs, electrolyte imbalances, tachyarrhythmias (Hunt et al., 2005). However, the common denominator for them it is that they create the metabolic and electrical conditions of the myocardium that are conducive to this type of arrhythmia takes place (Goldberger and Gold-berger, 1981). Finally, VF does not necessarily respond to the same electrophysiological mechanisms that cause VT, although as a general rule, a VF is always preceded by a VT. Electrocardiographic characteristics of potential malignancy in a VT preceding a VF, are: Polymorphism, R/T phenomenon (R on T) and a very high heart rate. If the VT is sustained, then it may be converted to VF which results fast and irregular episodes in ECG (Goldberger and Gold-berger, 1981; Hunt et al., 2005). In this way, CUDB database has the mixed rhythm annotations as both VT and VF in an ECG frame. Due to this reason, the specificity value is higher than sensitivity for the classification of VT and VF episodes.

**Table 1:** Performance of LSSVM classifier using DTFT features of ECG signals of all databases.

**4 s**				**5 s**				**8 s**			
**VT vs. VF**											
Kernels	Acc (%)	Sen (%)	Spe (%)	Kernels	Acc (%)	Sen (%)	Spe (%)	Kernels	Acc (%)	Sen (%)	Spe (%)
Linear	73.94	69.16	75.17	Linear	72.17	64.86	74.02	Linear	72.54	65.15	74.43
RBF	82.36	81.38	82.82	RBF	84.30	82.02	85.26	RBF	83.41	82.19	83.88
**NON-VF vs. VF**											
Kernels	Acc (%)	Sen (%)	Spe (%)	Kernels	Acc (%)	Sen (%)	Spe (%)	Kernels	Acc (%)	Sen (%)	Spe (%)
Linear	74.58	74.59	74.58	Linear	74.06	74.89	73.32	Linear	75.44	75.40	75.54
RBF	83.75	85.20	82.46	RBF	82.66	83.74	81.73	RBF	82.84	83.82	82.05
**NON-SHOCK vs. SHOCK**											
Kernels	Acc (%)	Sen (%)	Spe (%)	Kernels	Acc (%)	Sen (%)	Spe (%)	Kernels	Acc (%)	Sen (%)	Spe (%)
Linear	69.04	69.29	68.35	Linear	67.12	67.92	66.41	Linear	70.30	70.92	69.75
RBF	80.61	82.51	78.98	RBF	80.99	82.61	79.57	RBF	80.15	82.12	78.54

**Table 2:** Performance of RBF kernel LSSVM classifier using DTFT features of ECG signals of VFDB database.

**4 s**				**5 s**				**8 s**			
**VT vs. VF**											
Kernels	Acc (%)	Sen (%)	Spe (%)	Kernels	Acc (%)	Sen (%)	Spe (%)	Kernels	Acc (%)	Sen (%)	Spe (%)
RBF	79.19	80.98	78.05	RBF	77.22	79.72	76.00	RBF	82.83	79.64	84.27
**NON-VF vs. VF**											
Kernels	Acc (%)	Sen (%)	Spe (%)	Kernels	Acc (%)	Sen (%)	Spe (%)	Kernels	Acc (%)	Sen (%)	Spe (%)
RBF	89.05	85.81	92.97	RBF	89.44	86.58	92.81	RBF	89.81	86.38	93.97
**NON-SHOCK vs. SHOCK**											
Kernels	Acc (%)	Sen (%)	Spe (%)	Kernels	Acc (%)	Sen (%)	Spe (%)	Kernels	Acc (%)	Sen (%)	Spe (%)
RBF	83.52	82.84	86.26	RBF	83.63	81.87	85.67	RBF	84.26	83.38	85.25

**Table 3:** Performance of RBF kernel LSSVM classifier using DTFT features of ECG signals of CUDB database.

**4 s**				**5 s**				**8 s**			
**VT vs. VF**											
Kernels	Acc (%)	Sen (%)	Spe (%)	Kernels	Acc (%)	Sen (%)	Spe (%)	Kernels	Acc (%)	Sen (%)	Spe (%)
RBF	94.80	82.41	95.44	RBF	94.68	81.08	95.28	RBF	94.32	72.48	95.53
**NON-VF vs. VF**											
Kernels	Acc (%)	Sen (%)	Spe (%)	Kernels	Acc (%)	Sen (%)	Spe (%)	Kernels	Acc (%)	Sen (%)	Spe (%)
RBF	81.35	87.33	77.23	RBF	78.84	81.47	76.65	RBF	79.30	81.05	77.77
**NON-SHOCK vs. SHOCK**											
Kernels	Acc (%)	Sen (%)	Spe (%)	Kernels	Acc (%)	Sen (%)	Spe (%)	Kernels	Acc (%)	Sen (%)	Spe (%)
RBF	81.55	92.24	75.30	RBF	81.72	87.02	77.89	RBF	80.27	84.15	77.22

## 4. Discussion

The present work is based on the use of DTFT for extraction of ECG features. The performance of Taylor-Fourier features is compared with some of the existing features for detection of life threatening VA and the comparison result is shown in Table 4. In this work, the comparison of the performance of proposed method with existing approaches using the 8 s ECG features from CUDB database is shown. Here, for comparison, the performance of TCI, VFF, SPEC, CPLX, PSR features are also evaluated using CUDB database. Thakor et al. (1990) have proposed TCI features and threshold-based sequential detection algorithm for the classification of VT and VF. They have used 85 VF and 85 polymorphic and monomorphic VT episodes. They have evaluated the performance TCI features from 1 to 7 s ECG segments using threshold-based sequential detection algorithm. From their study, they have reported higher sensitivity, and specificity values for TCI feature from 6 and 7 s ECG segments. Similarly, Zhang et al. (1999) have evaluated the complexity measure from the ECG segments of different length for the classification of NSR, VT, and VF episodes. It has been reported that the complexity measure features of 5, 6, and 7 s ECG episodes have higher performance for the detection of VT and VF episodes. (Amann et al., 2007) have evaluated time delay features based on the PSR of ECG signal. The performance of the PSR based features of ECG signals from CUDB database and other databases is assessed using a threshold based classification approach for the detection of VF. They have also compared the performance of TCI, VFF, SPEC, CPLX, and PSR features from 8 s ECG episodes. It is evident from Table 4 that the existing algorithms for classification of VF and non-VF based on the analysis of 8 s ECG segments have less sensitivity value. However, the proposed Taylor-Fourier magnitude and phase features with LSSVM classifier has sensitivity value of 84.15% which is higher than the existing approaches. Similarly, by using VFDB database, the sensitivity and specificity values are 86.38 and 93.97%, which are higher as compared to the performance of Taylor-Fourier features of CUDB database. The specificity value of the proposed method is higher than those of the performance of few existing features such as the TCI, the CPLX and PSR based features. On the other hand, Alonso-Atienza et al. (2014) have compared the performance of TCI, VFF, SPEC, CPLX, and PSR features of 8 s ECG episodes with SVM classifier for the classification of VF vs. Non-VF and shock vs. Non-shock. They have reported (sensitivity, specificity) values of (49, 68%), (73, 89%), (74, 85%), and (23, 47%) using TCI, VFF, PSR, and CPLX features for the classification of VF vs. Non-VF episodes. Moreover, for the classification of shock vs. non-shock, the (sensitivity, specificity) values of (25, 37%), (65, 76%), (82, 93%) and (85, 92%) using CPLX, TCI, VFF, and PSR features of ECG signals from VFDB, CUDB databases. In the proposed method, the (sensitivity, specificity) values of LSSVM classifier using Taylor-Fourier magnitude and phase features from 4, 5, and 8 s ECG episodes are (85.81, 92.97%), (86.58, 92.81%), and (86.38, 93.97%), respectively. It has been observed that the accuracy value is improved with an increase in the duration of ECG segments. The CUDB database has mixed rhythm annotations for VT and VF cases. Therefore the performance of Taylor-Fourier features is less by using 8 s ECG segments.

**Table 4:** Comparison result for the classification of non-VF and VF.

**Features used**	**Sen (%)**	**Spe (%)**
Threshold Crossing Interval (TCI) (Thakor et al., 1990)	71.00	70.50
VF-Filter Algorithm (VFF) ()	30.00	99.50
Spectral Algorithm (SPEC) (Barro et al., 1989)	29.00	99.30
Complexity Measure Algorithm (CPLX) (Zhang et al., 1999)	56.40	86.60
Phase Space Representation (PSR) (Amann et al., 2007)	70.20	89.30
Taylor-Fourier Magnitude and phase (TFMP) (VFDB database)	86.38	93.97
Taylor-Fourier Magnitude and phase (TFMP) (CUDB database)	84.15	77.22

We have also compared the performance of proposed method with wavelet-based techniques for the detection of life-threatening ventricular arrhythmia episodes. Namarvar and Shahidi (2004) have used wavelet-singular value decomposition (SVD) based analysis of ECG and SVM classifier for the classification of VT and VF. They have tested their algorithm using cleaned and noisy ECG signals and reported a sensitivity and specificity values of 92 and 75% for the classification of VT and VF using features of cleaned ECG signal. In another study, Balasundaram et al. (2013) have applied Eigen decomposition using SVD on the wavelet coefficients matrix of ECG to evaluate features. They have considered linear discriminant analysis (LDA) classifier for the classification of VT and non-VT episodes and reported an overall accuracy of 97.10%. However, they have only used 63 number of 4 s ECG segments from 24 subjects for analysis. OH et al. (2017) have used discrete wavelet transform of ECG and various non-linear analysis methods with K-nearest neighbor (KNN) classifier for the classification of shock and non-shock episodes. They have reported 94.79 and 98.74% using features from 5 s ECG episodes. Our proposed method has higher performance regarding sensitivity and specificity values as 81.08 and 95.28% for the classification of VT and VF episodes as compared to the approach in Namarvar and Shahidi (2004). The approach reported by Balasundaram et al. (2013) has used less ECG instances as compared to the proposed method. Moreover, OH et al. (2017) have considered only shock vs. non-shock classification scheme and their approach have better performance as compared to our method. However, they have not considered VF vs. non-VF and VT vs. VF episodes. The wavelet-based methods use predefined basis functions which are implemented as finite impulse response (FIR) filters at different decomposition levels for the evaluation of wavelet coefficients. However, the proposed approach is simple, and it is based on the multiplication of ECG signal with Taylor-Fourier basis matrix for the evaluation of the Taylor-Fourier coefficients.

The advantages of the present work are written as follows.

- The method uses digital Taylor Fourier Transform of ECG signal for the evaluation of new diagnostic features.
- The method is evaluated using the Taylor Fourier magnitude and phase features from 4, 5, and 8 s ECG instances.
- The classification tasks such as shock vs. non-shock, VF vs. Non-VF and VT vs. VF are performed using the LSSVM classifier.
- Due to the presence of abnormal patterns with higher temporal variability, the mean values of phase features are high for VF class.
- The Taylor-Fourier features can be used for the detection of other pathologies from ECG signals.

The proposed work is evaluated using the ECG signals from only 57 subjects. More number of subjects can be used for assessing the performance of the proposed method. More features can be evaluated based on the non-linear analysis of both magnitude and phase parts of Taylor-Fourier coefficents of different modes of ECG signal for the detection of life-threatening ventricular arrhythmia. The complexity and burden for Taylor-Fourier transform have been discussed and compared with Fast Fourier Transform (FFT) in Zamora et al. (2017). Further computation reduction is achieved due to the fact that the Taylor-Fourier matrix only contains the frequencies of concern (Zamora et al., 2017). That is, matrices **B** and its pseudo inverse (**B**^†^) are computed just once, only those columns corresponding to the Taylor-Fourier filters with central frequencies between 0 and 45 Hz (varying each 5 Hz), are taken into account. This allows reducing the size of **B** from *CN*x*CN* to *CN*x*C*_*f*_*i*__, without affect the feasibility of the Taylor-Fourier transform method.

## 5. Conclusions

This paper has demonstrated the use of Taylor-Fourier filters for extracting the diagnostic features from ECG signal. The combination of Taylor-Fourier magnitude and phase features and LSSVM classifier has been used for detection of various life threatening arrhythmia. The Taylor-Fourier magnitude and phase feature have successfully quantify the pathological changes in ECG during life threatening VA and these features have different mean values for VF and non-VF class. The proposed method has higher sensitivity than those of the existing approach for detection of VF. More robust features from the magnitude and phase of Taylor-Fourier coefficients can also be evaluated for detection of life threatening VA and other heart ailments.

## Ethics statement

This study involved the human subjects. However, the data for the present study was taken from readily available public databases and hence, ethical statements associated with the study is not included.

## Author contributions

RT performed all data analysis and wrote the manuscript. AZ-M, JdlOS, MA, and JA advised the analysis and edited the manuscript. MA and JA conceptualized the experiment. MA and GN supervised the study, advised the analysis, and edited the manuscript.

### Conflict of interest statement

The authors declare that the research was conducted in the absence of any commercial or financial relationships that could be construed as a potential conflict of interest.

## Footnotes

^1^https://physionet.org/physiobank/database/cudb/

^2^https://physionet.org/physiobank/database/vfdb/
